# Generation and Function of Induced Regulatory T Cells

**DOI:** 10.3389/fimmu.2013.00152

**Published:** 2013-06-19

**Authors:** Erica G. Schmitt, Calvin B. Williams

**Affiliations:** ^1^Section of Rheumatology, Department of Pediatrics, Medical College of Wisconsin, Milwaukee, WI, USA

**Keywords:** Treg cells, Treg stability, immunotherapy, Treg function, gene expression profiling, TCR repertoire

## Abstract

CD4^+^ CD25^+^ Foxp3^+^ regulatory T (Treg) cells are essential to the balance between pro- and anti-inflammatory responses. There are two major subsets of Treg cells, “natural” Treg (nTreg) cells that develop in the thymus, and “induced” Treg (iTreg) cells that arise in the periphery from CD4^+^ Foxp3^−^ conventional T cells and can be generated *in vitro*. Previous work has established that both subsets are required for immunological tolerance. Additionally, *in vitro*-derived iTreg cells can reestablish tolerance in situations where Treg cells are decreased or defective. This review will focus on iTreg cells, drawing comparisons to nTreg cells when possible. We discuss the molecular mechanisms of iTreg cell induction, both *in vivo* and *in vitro*, review the Foxp3-dependent and -independent transcriptional landscape of iTreg cells, and examine the proposed suppressive mechanisms utilized by each Treg cell subset. We also compare the T cell receptor repertoire of the Treg cell subsets, discuss inflammatory conditions where iTreg cells are generated or have been used for treatment, and address the issue of iTreg cell stability.

## Introduction

Early insights into the existence of a subset of T cells capable of exhibiting dominant tolerance, or suppression of other cells in a paracrine manner, came from work done in neonatal thymectomy models. Neonatal thymectomy of newborn mice between days 2 and 4 of life resulted in various organ-specific T cell-mediated autoimmune diseases that could be prevented by CD4^+^ CD25^+^ T cells (Nishizuka and Sakakura, 1969; Sakaguchi et al., 1982, 1995; Asano et al., 1996). The discovery of mutations in the X chromosome-encoded gene *Foxp3* in human patients suffering from immune dysregulation, polyendocrinopathy, enteropathy, X-linked (IPEX) syndrome and in the mutant *scurfy* mice led to recent advances in regulatory T (Treg) cell biology (Chatila et al., 2000; Bennett et al., 2001; Brunkow et al., 2001; Wildin et al., 2001). Expression of the forkhead/winged helix transcription factor Foxp3 ultimately identifies Treg cells and is essential for the acquisition of suppressive function (Lin et al., 2007; Zheng and Rudensky, 2007). Conditional deletion of Foxp3 via retroviral expression of Cre in mature Treg cells resulted in the loss of Treg cell suppressive function and the gain of effector T cell properties, suggesting that continuous expression of Foxp3 is required for maintenance of the Treg cell phenotype (Williams and Rudensky, 2007). Furthermore, in a system where Treg cells express the human diphtheria toxin receptor, chronic diphtheria toxin-mediated ablation of Treg cells resulted in death from lympho- and myeloproliferative disease, confirming the continued need for Treg cells throughout the lifespan of normal mice (Kim et al., 2007).

These CD4^+^ CD25^+^ Foxp3^+^ Treg cells, which account for ∼10% of peripheral CD4^+^ T cells, are essential to the balance between pro- and anti-inflammatory responses at mucosal surfaces. There are two subsets of Treg cells, “natural” Treg (nTreg) cells and “induced” Treg (iTreg) cells. While nTreg cells develop as a distinct lineage in the thymus, iTreg cells arise from peripheral naïve conventional T (Tconv) cells and can be generated *in vitro* (Curotto de Lafaille and Lafaille, 2009). The focus of this review is iTreg cells, their mechanisms of generation, transcriptional profiles, TCR repertoires, potential for immunotherapy, and their stability *in vivo*.

## *In vivo* and *In vitro* Generation of iTreg Cells

CD4^+^ Tconv cells isolated from lymphoid organs and peripheral blood can be induced to express Foxp3 *in vitro* by T cell activation in the presence of TGF-β1 and IL-2 (Chen et al., 2003; Davidson et al., 2007). Following these important observations, several studies documented the development of functionally suppressive iTreg cells *in vivo*, either in a tolerogenic setting or arising during inflammation (Table 1). The emergence of iTreg cells has been observed in cases where antigens are encountered in the absence of optimal costimulation. This includes antigen delivery through intravenous injection (Thorstenson and Khoruts, 2001) and continuous infusion minipumps (osmotic pumps) (Apostolou and von Boehmer, 2004), or by the administration of non-depleting anti-CD4 antibodies (Cobbold et al., 2004). Oral administration of antigen leads to the development of iTreg cells that are functionally suppressive in a mouse model of asthma and are required to establish oral tolerance (Mucida et al., 2005; Curotto de Lafaille et al., 2008). Suboptimally activated dendritic cells support iTreg cell development. For example, dendritic cells targeted with low dose antigens by anti-DEC-205 (dendritic and epithelial cells, 205 kDa, multilectin endocytic receptor) antibodies (Kretschmer et al., 2005) and tolerogenic dendritic cells, residing in the small intestine lamina propria and mesenteric lymph node (Coombes et al., 2007; Sun et al., 2007), promote iTreg cell generation. In addition, several studies have demonstrated that the commensal microbiota contribute to iTreg cell development (Round and Mazmanian, 2010; Atarashi et al., 2011; Geuking et al., 2011). Alternatively, iTreg cells can be generated during states of chronic inflammation. Examples where chronic inflammation may support iTreg development include mouse models of asthma (Curotto de Lafaille et al., 2008; Weiss et al., 2012), colitis that occurs during T cell expansion in a lymphopenic environment (Haribhai et al., 2009), adoptive transfer immunotherapy for the treatment of Foxp3-deficiency (Haribhai et al., 2011), and infection with intestinal parasites (Grainger et al., 2010). The extent of iTreg cell development in locations other than mucosal tissues is not as well documented. However, recently iTreg cell development has been demonstrated to occur locally in immune privileged sites such as the spinal cords of mice with experimental autoimmune encephalomyelitis (Weiss et al., 2012) and in the eye (Zhou et al., 2012; McPherson et al., 2013). Tissue-specific Foxp3 induction has also been demonstrated in response to a neo-self antigen restricted to the pancreas (Thompson et al., 2011). These data generally support the biological relevance of the mechanisms that generate and sustain iTreg cells.

**Table 1:** **Models generating *in vivo*-derived iTreg cells**.

Model	Time point	Location	% of CD4^+^ cells that are iTreg cells	Reference
IV injection of low dose peptide antigen following transfer of CD4^+^ T cells from RagKO TCR transgenic mice into unirradiated BALB/c mice	8 days after IV injection of Ag	Spleen	∼20–25	Thorstenson and Khoruts (2001)
Peptide delivery via osmotic pump implanted in RagKO TCR transgenic mice	14 days after implant of continuous delivery system	Spleen	∼20–25	Apostolou and von Boehmer (2004)
Non-depleting anti-CD4 antibodies during skin grafting onto RagKO TCR transgenic mice	7 days after challenge with second graft	Skin graft	∼50	Cobbold et al. (2004)
Homeostatic proliferation after transfer of Tconv cells into T-B monoclonal mice	1 month	Peripheral blood	∼10	Curotto de Lafaille et al. (2004)
Antigen delivery to dendritic cells using anti-DEC-205 antibodies following transfer of CD4^+^ T cells from RagKO TCR transgenic mice	14 days post injection	Pooled spleen, MLN, inguinal LN	∼15	Kretschmer et al. (2005)
Oral tolerance established in a model of allergic airway inflammation using T-B monoclonal mice; oral OVA followed by immunization and intranasal challenge	2 days post intranasal challenge	Lung and BAL	∼10	Mucida et al. (2005), Curotto de Lafaille et al. (2008)
T cell transfer model of colitis	∼100 days post induction	MLN	∼9	Haribhai et al. (2009)
Establishment of oral tolerance after transfer of CD4^+^ T cells from RagKO TCR transgenic mice during helminth infection	7 days post infection	MLN and Peyers patch	∼50	Grainger et al. (2010)
Treatment of Foxp3-deficiency with nTreg plus Tconv cells	50-day-old mice	PLN	∼1	Haribhai et al. (2011)
Transfer of CD4^+^ T cells from RagKO TCR transgenic mice to RagKO mice expressing the cognate antigen in the pancreas	Diabetes onset	Pancreatic LN	∼20	Thompson et al. (2011)
MCA-38 colon adenocarcinoma tumor	2 weeks post tumor injection	Tumor infiltrating lymphocytes	∼45	Weiss et al. (2012)
Experimental autoimmune encephalomyelitis – chronic stage	20–30 days post EAE onset	Spinal cord	∼10–15	Weiss et al. (2012)
Transfer of retinal protein specific CD4^+^ T cells from RagKO TCR transgenic mice to the eyes of WT hosts	8 days post injection	Eye	∼30	Zhou et al. (2012)

*This table documents model systems where *in vivo*-derived iTreg cells form, the time point and location that the iTreg cells were observed, the percentage of CD4^+^ T cells that developed into iTreg cells, and the reference. BAL, bronchoalveolar lavage; EAE, experimental autoimmune encephalomyelitis; MLN, mesenteric lymph node; OVA, ovalbumin; pLN, peripheral lymph node*.

Multiple signaling pathways converge to influence the efficiency of iTreg cell generation. Specific TCR affinity and TCR-derived signals, costimulatory molecules, and cytokines promote optimal *in vivo* iTreg cell development. Low doses of high affinity ligands promote iTreg cell generation by creating a decreased aggregate TCR stimulation as compared to Tconv cells (Kretschmer et al., 2005; Gottschalk et al., 2010). Strong CD28 costimulation (Semple et al., 2011) and CTLA-4 blockade (Zheng et al., 2006) are detrimental to *de novo* induction of Foxp3 whereas activation of Tconv cells under conditions of suboptimal costimulation promotes the induction of Foxp3. Furthermore, signaling via the programed death (PD) 1-PD-ligand (PD-L) pathway promotes both the induction and maintenance of iTreg cells (Francisco et al., 2009). TCR-dependent activation of the PI3K-AKT-mTOR axis is an important negative regulator of peripheral Treg cell differentiation. AKT inhibits Foxo proteins, which normally facilitate Foxp3 induction (Kerdiles et al., 2010; Ouyang et al., 2010). Therefore, enhancing AKT signaling, either by overexpression (Haxhinasto et al., 2008) or by deletion of negative regulators of AKT, such as phosphatase and tensin homolog (PTEN) (Sauer et al., 2008) or the E3 ubiquitin ligase Cbl-b that degrades the regulatory subunit of PI3K (Wohlfert et al., 2006; Harada et al., 2010), adversely impacts iTreg cell development. Alternatively, inhibition of PI3K or mTOR enhances iTreg cell development (Battaglia et al., 2005; Sauer et al., 2008). Blockade of signals through the C3aR and C5aR complement receptors also decreases signaling through the PI3K-AKT-mTOR pathway thereby enhancing autoinductive signaling by TGF-β1 to generate iTreg cells (Strainic et al., 2013).

Both TGF-β1 and IL-2 are required for iTreg cell induction. TGF-β1 signaling promotes the binding of NFAT and Smad3 to the conserved non-coding sequence-1 (CNS1) enhancer and ultimately stimulates histone acetylation and Foxp3 induction (Tone et al., 2008). These data are further supported by the observation that CNS1 deletion impairs iTreg cell generation in gut-associated lymphoid tissues (Zheng et al., 2010). TGF-β1 also limits DNA methyltransferase I recruitment to the Foxp3 locus, a molecule that normally functions to prohibit promiscuous Foxp3 induction after TCR stimulation (Josefowicz et al., 2009). IL-2 is likewise required for iTreg generation *in vitro* (Davidson et al., 2007). *In vivo*, IL-2 has a role in Treg cell survival (D’Cruz and Klein, 2005), proliferation (Fontenot et al., 2005a), and stability (Chen et al., 2011) therefore a role for *in vivo* induction has been more difficult to parse out. Perhaps in support of a role for induction, cells in the periphery that are poised to develop into iTreg cells require only IL-2 for Foxp3 induction (Schallenberg et al., 2010). IL-2 also functions to limit the polarization of activated CD4^+^ T cells into the Th17 lineage (Laurence et al., 2007). Similar to IL-2, all-trans retinoic acid restricts reciprocal Th17 polarization (Xiao et al., 2008). CD103^+^ gut-derived tolerogenic dendritic cells, which play an important role in the generation of iTreg cells serve as a source of retinoic acid (Coombes et al., 2007). Activation of the aryl hydrocarbon receptor by the ligands 2,3,7,8-tetrachlorodibenzo-*p*-dioxin and 2-(1’H-indole-3’-carbonyl)-thiazole-4-carboxylic acid methyl ester supports the generation of functional, stable iTreg cells by promoting both the generation of retinoic-acid producing tolerogenic dendritic cells and demethylation of the Foxp3 promoter (Quintana et al., 2008, 2010; Singh et al., 2011). In summary, antigenic TCR stimulation with low dose/high affinity ligands, suboptimal costimulation, TGF-β1, IL-2, and retinoic acid all facilitate the induction of Foxp3 expression in peripheral CD4^+^ Tconv cells *in vivo*.

## Transcriptional Landscape and Function of iTreg Cells Versus nTreg Cells

The pivotal role of the X-linked gene *Foxp3* in the identity of a Treg cell prompted investigation into the Foxp3-dependent and -independent programs of the Treg cell transcriptional signature. Mice possessing an altered Foxp3 locus, in which DNA encoding EGFP was inserted in frame into exon 11 at the C-terminal end of the Foxp3 locus (*Foxp3*^Δ*EG*F*P*^), express a non-functional ΔFoxp3-EGFP fusion protein that is devoid of the nuclear localization sequence and residues involved in DNA binding (Lin et al., 2007). In heterozygous *Foxp3*^Δ*EG*F*P*±^ female mice, which have random inactivation of one of the two X chromosomes, the frequency of thymocytes expressing the non-functional ΔFoxp3-EGFP fusion protein was similar to thymocytes expressing normal Foxp3. The EGFP^+^ cells from these mice also expressed several Treg cell-associated molecules, such as CD25, CTLA-4, GITR, and CD44, but were not suppressive and produced Th1- and Th2-associated cytokines. Many transcripts commonly found in Treg cells were identified by gene array in the EGFP^+^ cells, these included *Il2ra*, *Ctla4*, and *Itgae*. The expression of additional genes suggestive of a cytotoxic effector program, such as *Gzma*, *Gzmb*, and *Gzmk*, and genes encoding chemokine receptors such as *Cxcr6*, were also observed (Lin et al., 2007).

In a separate set of studies, cells destined to be Treg cells were marked with an in frame insertion of GFP into a Foxp3 locus disrupted by a stop codon. This resulted in Foxp3 transcription, but not translation, and also allowed for the separation of Foxp3-dependent and independent factors (Gavin et al., 2007). As a result, several characteristic Treg cell markers, such as CD25, CD44, CTLA-4, GITR, and ICOS, were found to be Foxp3-independent. Although several hallmark Treg cell markers were found, suppressive activity was lost in the absence of Foxp3 protein. These studies confirmed that Foxp3 suppressive function and stability are dependent on a functional Foxp3 protein. Together, they suggest that some aspects of commitment to the Treg cell lineage begin independently of a functional Foxp3 protein.

Fundamental work from Hill et al. (2007) combined gene expression profiles of Treg cells obtained under many different conditions and identified a canonical Treg cell signature. This study confirmed previous work, in that it identified Treg cell-associated genes that were not correlated with Foxp3 expression, but they also organized the Treg signature into several co-regulated gene clusters influenced by a defined set of factors. This Treg cell transcriptional signature provides a framework for comparison of Treg cells derived by alternative methods or in varying anatomical locations. Treg cells found in different anatomical locations within the same individual have unique TCR repertoires, variations in their cell surface phenotypes, and distinct gene expression profiles (Lathrop et al., 2008; Feuerer et al., 2010). These findings are consistent with the idea that subsets of Treg cells exist, and that Treg cell suppressive activity may be finely tuned to the microenvironment. Currently there is no consistent, reliable marker to distinguish nTreg and iTreg cells *in vivo*, although in some systems Helios (Thornton et al., 2010) and Neuropilin 1 (Nrp1) (Weiss et al., 2012; Yadav et al., 2012) have been suggested to specifically identify nTreg cells. Others have determined that expression of Helios, an Ikaros family transcription factor, results from more general T cell activation and proliferation (Akimova et al., 2011). Nrp1 is a receptor for TGF-β1 and has been reported to activate the latent form of TGF-β1 and promote Treg cell activity (Glinka and Prud’homme, 2008). Under homeostatic conditions, this marker seems to reliably distinguish nTreg cells from iTreg cells; however, iTreg cells present in inflammatory conditions can express Nrp1 (Weiss et al., 2012). The lack of a suitable surface marker has hampered the ability to effectively distinguish the characteristics of the two subsets in a host without using a transfer model to mark the populations.

Many studies have compared the transcriptional signatures of nTreg and iTreg cells in an attempt to distinguish the two subsets. Given that a portion of the Treg cell signature is Foxp3-independent, it was not surprising that the transcriptional signature of iTreg cells derived *in vitro* did not fully recapitulate the observed nTreg cell genetic signature (Haribhai et al., 2009; Feuerer et al., 2010). On the other hand, iTreg cells that were allowed to develop *in vivo* were more similar to nTreg cells than their *in vitro*-derived counterparts (Feuerer et al., 2010; Haribhai et al., 2011). However, nTreg cells and *in vitro*-derived iTreg cells that are stably maintained *in vivo* for approximately 3 months share similar transcriptional profiles (Schmitt et al., 2012). This included the expression of many genes associated with Treg cell suppressive function such as *Il2ra*, *Ctla4*, *Gzmb*, and *Il10*. Thus, the transcriptional signature of *in vitro*-derived iTreg cells and nTreg cells, although much different immediately after generation *in vitro*, converge as the *in vitro*-derived iTreg cells are selected and maintained *in vivo*. While the collective gene expression data suggest that the two Treg subsets share similar suppressive mechanisms, the observed requirement for both subsets in maintaining tolerance hints that distinct suppressive mechanisms that play discrete roles, either in different anatomical locations or in different types of inflammation, may yet be identified. Indeed, a recent study uncovered four “Treg cell-representative regions” which included regions of *Foxp3*, *Tnfrsf18*, *Ctla4*, and *Ikzf4* that display demethylation patterns in nTreg cells that are distinct from those observed in Tconv and iTreg cells. This nTreg cell-specific methylation pattern is instrumental in establishing Treg cell-type gene expression (Ohkura et al., 2012). Additionally, recent work demonstrated an important role for Foxo1 in establishing the Foxp3-independent Treg cell transcriptional program, in part by inhibiting IFN-γ expression in Treg cells (Ouyang et al., 2012).

The interaction of Foxp3 with several different molecules is important for Treg cell transcriptional activity. The Foxp3 gene has numerous structural domains including a transcriptional repression domain at the N-terminus, followed by a zinc finger domain, a leucine zipper domain, and a forkhead DNA binding domain. A series of serendipitous discoveries using a *Foxp3*^*G*F*P*^ (*Foxp3^*tm2Ayr*^*) fusion protein to mark Treg cells, in which GFP is fused to the amino terminus of Foxp3 (Fontenot et al., 2005b), revealed altered autoimmune disease phenotypes. The *Foxp3*^*G*F*P*^ fusion protein reduces or eliminates the interaction of the N-terminal domain of the Foxp3 gene with Eos, Tip60, HDAC7, and HIF-1α; however, distal interactions with NFAT, AML1/Runx-1, RORα, and IRF4 are maintained or enhanced. As a result, the transcriptional activity of Treg cells was altered leading to accelerated type 1 diabetes in disease prone NOD mice (Bettini et al., 2012) while protecting mice from autoimmune arthritis in the K/BxN model (Darce et al., 2012).

Much work has been done to uncover the molecular mechanisms of Treg cell suppressive activity delineated by the transcriptional data. However, there have been few attempts to discriminate between the two subsets. Consequently, with regard to the specific mechanisms utilized to control inflammation, the “division of labor” between nTreg cells and iTreg cells remains largely unresolved (Curotto de Lafaille and Lafaille, 2009). In general, Treg cell suppression has been demonstrated to modify effector cell activity at several different stages within the immune response (Suri-Payer et al., 1998). Suppression by Treg cells can operate at the early stages, by limiting cell activation and proliferation. Initial studies using *in vitro* proliferation assays demonstrated the ability of Treg cells to control effector cell proliferation in an IL-2 dependent manner (Thornton and Shevach, 1998). Gene expression profiling of the suppressed CD4^+^ T cells subsequently showed the induction of genes involved in growth arrest or the inhibition of proliferation (Sukiennicki and Fowell, 2006). In the later stages of the immune response, Treg cells have been shown to control effector cell differentiation and function in the target tissues (Oldenhove et al., 2003; Sarween et al., 2004; DiPaolo et al., 2005). The ability of Treg cells to effectively control diverse types of inflammation has been associated with Treg cell upregulation of specific transcription factors (Campbell and Koch, 2011). Treg cell expression of T-bet, IRF4, and STAT3 contribute to the ability of Treg cells to control the associated Th1 (Koch et al., 2009), Th2 (Zheng et al., 2009), and Th17 (Chaudhry et al., 2009) polarized inflammation, respectively. In addition, Treg cell expression of GATA-3 is important for their accumulation at the site of inflammation as a Treg cell-specific deletion of GATA-3 led to a failure of Treg cell accumulation in tissues and the acquisition of effector cytokine production (Wohlfert et al., 2011). Thus, it appears that Treg cells possess the ability to express transcription factors associated with the type of inflammation they are controlling, which in turn provides them with the ability to adapt their suppressive program to the surroundings.

**Figure 1:**
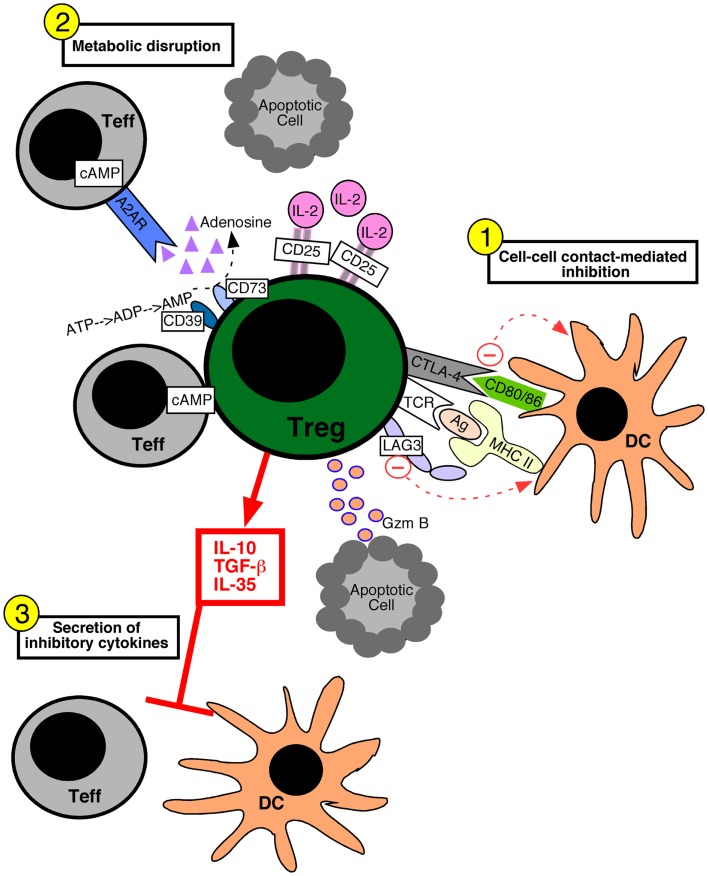
**Mechanisms of regulatory T cell-mediated suppression**. Regulatory T (Treg) cells can utilize several different suppressive mechanisms falling into three broad categories: (1) cell–cell contact-mediated suppression, (2) the metabolic disruption of effector T (Teff) cells, and (3) the secretion of inhibitory cytokines. (1) Contact-mediated suppression dampens the immunostimulatory properties of dendritic cells (DC) and occurs via the engagement of Treg cell inhibitory receptors such as CTLA-4 and LAG-3 with CD80/86 and MHC molecules on the DC, respectively. Delivery of granzyme B (Gzm B) to Teff cells leads to apoptosis. (2) Metabolic disruption of effector T cells is mediated by Treg cell delivery of cAMP to effector T cells via gap junctions, the generation of adenosine by the Treg cell ectoenzymes CD39 and CD73 which acts on Teff cell adenosine receptors (A2AR), and by Treg cell consumption of IL-2 thereby depriving Teff cells of growth factors. (3) Treg cells secrete inhibitory cytokines such as IL-10, IL-35, and TGF-β1, which inhibit both T cells and DCs.

Various molecular mechanisms of Treg cell-mediated suppression have been proposed. These suppressive mechanisms fall into three broad categories: suppression mediated by cell–cell contact, metabolic disruption, and the secretion of inhibitory cytokines (Figure 1). Cell–cell contact suppression operates via molecules such as CTLA-4 (Wing et al., 2008) and LAG-3 (Liang et al., 2008), which may function to modulate the immunostimulatory capacity of dendritic cells. In addition, Treg cells secrete cytotoxic molecules such as Granzyme B, which is presumed to require cell–cell contact (Grossman et al., 2004; Gondek et al., 2005). Metabolic disruption can occur via the delivery of cAMP to effector T cells through gap junctions (Bopp et al., 2007). The ectoenzymes CD39 and CD73 on Treg cells generate adenosine, which binds the adenosine receptor 2A on effector T cells and increases intracellular cAMP to suppress their function (Deaglio et al., 2007). Lastly, the increased constitutive expression of CD25 on Treg cells may allow them to out-compete effector cells for the growth factor IL-2, leading to cytokine deprivation-induced apoptosis of the effector T cells (de la Rosa et al., 2004; Pandiyan et al., 2007). Inhibitory cytokines such as TGF-β1 (Powrie et al., 1996), IL-35 (Collison et al., 2007), and IL-10 (Asseman et al., 1999) have been implicated in Treg cell suppressive function, and may serve to specifically dampen the activation of antigen presenting cells or inhibit effector T cell proliferation.

The immunomodulatory cytokine IL-10 has been studied extensively in relation to Treg cell biology. IL-10 is particularly important for Treg cells at environmental interfaces, as a Treg cell-specific inactivation of IL-10 results in spontaneous colitis, heightened immune-mediated lung hyperreactivity, and increased skin sensitivity (Rubtsov et al., 2008). Treg cell-derived IL-10 controls Th17 cells and a unique population of T cells displaying features of both Th1 and Th17 cells (Th1 + Th17) in a transfer model of colitis (Huber et al., 2011). In a model where Foxp3-deficiency was treated with nTreg cells plus *in vivo*-derived iTreg cells, gene expression profiling revealed that both Treg cell types over-expressed *Il10* as compared to naïve Tconv cells, suggesting a possible role for IL-10 as an iTreg cell mechanism of suppression (Haribhai et al., 2011). Recently, it was demonstrated that IL-10 produced by iTreg cells could replace nTreg cell-derived IL-10 in the cure of experimental colitis. Reversal of the experimental conditions was similarly effective, defining the novel principle of reciprocal compensation between Treg cell subsets, which was necessary to establish tolerance in this model (Schmitt et al., 2012). This work also demonstrated that iTreg cells limited the frequency of ex-iTreg cells adopting a Th1, Th17, or Th1 + Th17 cell fate, in concordance with previous data looking at the function of nTreg cells (Huber et al., 2011). Thus, it is possible that under certain circumstances, both Treg subsets must possess the ability to operate via the same mechanism. Further studies are needed to determine whether the principle of reciprocal compensation is model-specific or can be globally applied in situations where a known Treg cell defect exists.

## T Cell Receptor Repertoire of the Treg Cell Subsets

In the thymus, developing Tconv cells and nTreg cell precursors have unique affinity requirements (Jordan et al., 2001; Apostolou et al., 2002). Induction of Foxp3 requires an agonist self-peptide, and the frequency of nTreg cells that develop is directly proportional to the strength of the signal (Relland et al., 2009). Furthermore, autoreactivity of the nTreg cell compartment has been demonstrated, despite a normal response to central tolerance mechanisms (Romagnoli et al., 2002; Hsieh et al., 2004). Given the observed bias of nTreg cells to self antigen, several studies have sought to compare the TCR repertoires of nTreg and Tconv cells. Studies that have reported differences between nTreg and Tconv repertoires have analyzed the TCRα complementarity determining region (CDR3) of mice with fixed transgenic TCRβ chains and a restricted *Tcra* locus. In these reports, the nTreg and Tconv TCR repertoires were found to be equally diverse, however the degree of observed overlap between the two populations varied (Hsieh et al., 2004, 2006; Pacholczyk et al., 2006, 2007; Wong et al., 2007b). In a separate system with limited diversity, the repertoire of the nTreg cells responding to a foreign antigen was found to be more limited and clonally distinct compared to Tconv cells also responding to the antigen (Relland et al., 2012). In contrast to the self-specificity seen in the nTreg cell population, iTreg cells are thought to be specific for foreign antigen, given that the iTreg cell population is derived from the Tconv cell pool. Therefore, it was not surprising that the iTreg cell TCR repertoire shared minimal overlap with that of nTreg cells (Haribhai et al., 2011). This limited overlap may in part contribute to the requirement for both nTreg and iTreg cells in the resolution of autoimmune diseases, as the combination provides a more diverse TCR repertoire. Evidence from a handful of TCR repertoire studies suggests that iTreg formation in non-mucosal tissues, such as in the central nervous system of mice with experimental autoimmune encephalomyelitis (Liu et al., 2009) and in the pancreas of diabetic mice (Wong et al., 2007a), may be limited. Minimal TCR repertoire overlap was observed between Tconv and Treg cells at these locations, supporting a role for Treg cell recruitment rather than induction. Furthermore, mice that lack iTreg cells due to a genetic ablation of the intronic *Foxp3* enhancer CNS1 maintain tolerance to systemic and tissue-specific antigens but develop inflammation at the mucosal interfaces of the lung and gastrointestinal tract (Josefowicz et al., 2012). Interestingly, CNS1 deficient mice also display increased fetal resorption due to a lack of fetal alloantigen-specific iTreg cells (Samstein et al., 2012). These data support the notion that iTreg cell TCRs may function to expand tolerance to non-self antigens, particularly those present at mucosal interfaces.

To gain further insight into the TCR specificity of Treg cells, several groups have created TCR transgenic mice that harbor a TCR derived from a Treg cell (Bautista et al., 2009; Leung et al., 2009). Interestingly, nTreg cells were only efficiently generated when the transgenic cells were present at a low clonal frequency. These studies suggested that the development of nTreg cells is a saturable process that plateaus, most likely due to intraclonal competition for MHC/peptide complexes. However, recent work has demonstrated that a limited, fixed pool of *in vitro*-derived iTreg cells contains a large number of clones with TCRs that can be maintained within the iTreg cell niche, and mice receiving equivalent numbers of the same iTreg cells maintained distinct clones (Schmitt et al., 2012). This is in agreement with previous work demonstrating that high TCR diversity is important for the optimal function of Treg cells in a model of experimental acute GVHD (Fohse et al., 2011). In contrast to the nTreg cell niche, the iTreg cell niche is probably not constrained by the number of available antigens, given the proposed specificity for non-self and the complexity of the microbiome. This suggests that the size of the iTreg cell population may not be limited by TCR specificity, but may be determined by other factors such as the number of tolerogenic antigen presenting cells (Coombes et al., 2007), local concentrations of TGF-β1 (Marie et al., 2005; Li et al., 2006) and IL-2 (Chen et al., 2011), and signaling via the PD 1–PD-L pathway (Francisco et al., 2009). In addition, members of the Tumor Necrosis Factor Receptor superfamily expressed on Treg cells, including GITR (Ray et al., 2012) and OX40 (Piconese et al., 2010), have also been shown to be important for Treg cell proliferative fitness. Increased IL-2 signaling, via administration of IL-2 immune complexes or through constitutive STAT5b signaling, allows for Treg cell division in the absence of TCR signaling (Zou et al., 2012). During the treatment of autoimmune conditions, such as experimental colitis, high levels of IL-2 could allow for the maintenance of a diverse population of iTreg cells. Other cell types, such as IL-10-producing CXCR1^+^ macrophages in the lamina propria are important for Treg cell proliferation in the setting of oral tolerance, and may contribute to the size and composition of the iTreg cell population (Hadis et al., 2011). It is also likely that the nTreg cell subset dictates the size of the iTreg cell niche, because in the absence of nTreg cells, the iTreg cell compartment expands ∼fivefold (Haribhai et al., 2009). A recent study demonstrated that *in vitro*-derived iTreg cells cotransferred with naïve T cells into *Rag^−*/*−^* hosts were not effective in preventing colitis and many of the iTreg cells had decreased Foxp3, CTLA-4, and CD25 expression (Ohkura et al., 2012). Thus, cooperation between nTreg and iTreg cells, which is essential to establish tolerance, could therefore influence the composition of the iTreg cell niche. Manipulation of the factors implicated in shaping the iTreg cell niche may provide a mechanism to control the size, specificity, and/or function of the iTreg cell compartment.

## Immunotherapy with iTreg Cells

Statistics published by the National Institutes of Health indicate that chronic autoimmune disease affects ∼5–8% of the U.S. population, an estimated 14.7–23.5 million individuals, and the prevalence is rising (NIAID, 2005). Existing therapeutic approaches are inadequate and current research efforts must focus on restoring the balance between pro- and anti-inflammatory responses. A decrease in Treg cell numbers and/or function has been associated with many human autoimmune diseases (Long and Buckner, 2011). Currently, *ex vivo* expanded nTreg cells are being used in umbilical cord blood transplantation clinical trials, where the benefit to risk ratio is high due to the risk of life-threatening GVHD (Brunstein et al., 2011). Although nTreg cells were functionally suppressive *in vivo* after several rounds of stimulation and expansion, the optimal ≥1:1 nTreg to peripheral blood mononuclear cell ratio could not be achieved (Hippen et al., 2011b). Therefore iTreg cells, which can be generated in large numbers *ex vivo* and have been shown to operate in a xenogeneic model of GVHD, may offer an alternative to nTreg cells (Hippen et al., 2011a). The ability of iTreg cells to be generated in large numbers makes them an attractive alternative for the treatment of human autoimmune disorders unresponsive to current approaches (Trzonkowski et al., 2009; Brunstein et al., 2011; Di Ianni et al., 2011; Hippen et al., 2011a). *In vitro*-derived iTreg cells are functionally suppressive in animal models of inflammatory bowel disease (Fantini et al., 2006), diabetes (Weber et al., 2006), autoimmune gastritis (DiPaolo et al., 2007), experimental autoimmune encephalitis (Selvaraj and Geiger, 2008), and Foxp3-deficiency (Huter et al., 2008). Notably, *in vitro*-derived iTreg cells contribute to tolerance in disease models where *in vivo*-derived iTreg cells are absent (Haribhai et al., 2009, 2011). Moreover, iTreg cells can be used to augment and restore regulatory networks in situations where nTreg cells are exhausted or defective (Schmitt et al., 2012). Yet, in many of these models the specific Treg cell suppressive mechanism that is important and functional at an individual site of inflammation remains poorly understood. It is likely that iTreg cells can operate via multiple means but that particular suppressive mechanisms may vary in importance in each autoimmune disease or in different stages of the same disease.

A recent phase 1/2a clinical study conducted in 20 patients with refractory Crohn’s disease demonstrated a clinically significant effect of a single infusion of Treg cells in 40% of patients 5 weeks post-infusion (Desreumaux et al., 2012). Patients’ cells were expanded *in vitro* in response to ovalbumin (OVA) and cloned by limiting dilution to generate IL-10–producing OVA-specific Treg cells. Whether these cells are functioning purely as IL-10–producing T regulatory (Tr1) cells or as Foxp3^+^ iTreg cells is unclear, as ∼60% of the OVA-Treg cells expressed Foxp3. For human cells, *in vitro* activation leads to Foxp3 expression within 48 h, peaking at 4–6 days, and diminishing by 10–14 days post-activation, leaving only a fraction of cells Foxp3^+^(Pillai et al., 2007). Bonafide human Foxp3^+^ Treg cells can be identified by characteristic epigenetic changes within the Foxp3 locus (Baron et al., 2007), however, tracking of the transferred cells was not feasible in this case (Desreumaux et al., 2012). Regardless of this caveat, both Foxp3^+^ and Foxp3^−^ IL-10-producing regulatory cells can control pathogenic T helper cells in mouse models of intestinal inflammation (Huber et al., 2011). This initial clinical study provides the groundwork for additional research into adoptive transfer immunotherapy for autoimmune diseases refractory to current therapies.

Another issue with adoptive transfer immunotherapy is the *in vivo* stability of iTreg cells. In a model of experimental colitis, iTreg cells were recovered from successfully treated mice (Haribhai et al., 2009; Schmitt et al., 2012). Conversely, in a mouse model of GVHD these cells did not persist (Beres et al., 2011). Perhaps, the degree of ongoing inflammation will hamper the efficacy of these cells for therapy. If relevant clones could be pre-selected, enhancing the possibility that these cells will be expanded and/or maintained via interactions with their cognate ligand *in vivo*, this may increase the usefulness of iTreg cells. Further, excessive regulation may hamper normal immune responses to invading organisms, thus a fine balance between limiting disease progression and impeding natural responses to infectious agents needs to be established.

## Stability of iTreg Cells

The self-specificity of nTreg cell TCRs creates the potential for autoimmunity that is averted by stable Foxp3 expression. Several recent studies have scrutinized the stability of nTreg cells, both long term and in pro-inflammatory conditions. Indeed, there is some disparity in the reports regarding Treg cell plasticity. On one hand, both nTreg and iTreg cells were shown to convert to a pro-inflammatory Th17 phenotype in the presence of IL-6, IL-1, and TGF-β1 *in vitro* (Yang et al., 2008). These “exFoxp3” cells were also tracked in a study using Foxp3-GFP-Cre BAC transgenic mice bred to mice that expresses YFP from the *Rosa26* promoter after removal of a *lox*P-“stop” cassette (Rosa26-loxP-Stop-loxP-YFP). In this model, all cells that expressed Foxp3 at any time during their lifespan deleted the “stop” cassette and remained YFP^+^, thus marking “exFoxp3” cells with a YFP^+^ Foxp3^−^ phenotype. These “exFoxp3” cells produced pro-inflammatory cytokines, were pathogenic, and the TCR repertoire analysis suggested that they were derived from both nTreg and iTreg cells (Zhou et al., 2009). In contrast to this report, a group that used an inducible labeling system found the nTreg cell population to be stable throughout the lifespan of the mouse and in the setting of *Listeria* infection, lymphopenia, and autoimmune inflammation (Rubtsov et al., 2010). In this model, mice with a Foxp3-eGFP-Cre-ERT2 (ERT2, mutated human estrogen receptor ligand-binding domain) fusion protein were bred to mice in which the *Rosa26* locus contains a loxP site-flanked STOP cassette followed by YFP. The GFP-CreERT2 fusion protein is normally sequestered in the cytosol, but administration of tamoxifen allows nuclear localization and constitutive, heritable labeling of a cohort of Treg cells with YFP. The differences observed between these studies were attributed to the caveats with the BAC transgenic system, in which cells that transiently expressed Foxp3, prior to stabilization, would be labeled (Miyao et al., 2012). The latest labeling system revealed that mouse T cells can upregulate Foxp3 during activation (Miyao et al., 2012), as observed with human T cells (Pillai et al., 2007), and that this promiscuous Foxp3 expression accounts for the documented instability of the Treg cell lineage. Taken together, these results demonstrate that nTreg cells express Foxp3 in a stable, heritable fashion.

Analysis of the *Foxp3* locus revealed three intronic elements within the proximal CNS that influence the composition, stability, and size of the Treg cell compartment (Zheng et al., 2010). To determine the function of the CNS elements *in vivo*, individual deletions of each CNS element were created. These analyses revealed CNS1, which contains binding sites for NFAT, RAR/RXR, and Smad3, to be particularly important for the development of iTreg cells. In CNS1 knockout mice the efficiency of *in vivo* and *in vitro* generation of iTreg cells was reduced. CNS2 was shown to be important in the heritable maintenance of Foxp3. The CpG motifs in CNS2, also known as the Treg cell-specific demethylated region (TSDR), are demethylated in nTreg cells, but not in iTreg cells produced *in vitro* (Floess et al., 2007; Polansky et al., 2008) (Table 2). Interestingly, *in vitro*-derived iTreg cells that were stably maintained *in vivo* for ∼3 months could achieve at least partial demethylation of the TSDR (Schmitt et al., 2012). Treatment with inhibitors of DNA methyltransferases (Polansky et al., 2008; Lal et al., 2009) and histone deacetylases (Tao et al., 2007) can enhance the stability of Foxp3 expression. In a similar fashion, progesterone (Lee et al., 2012), rapamycin (Battaglia et al., 2005), and retinoic acid (Mucida et al., 2009) promote iTreg cell stability and/or generation, and could be incorporated into *in vitro* induction protocols to create stable iTreg cells for immunotherapy. After demethylation, a Foxp3-Runx-1-CBFb complex is recruited to CNS2 and may represent an important lineage specification event (Zheng et al., 2010). Since demethylation is required for the complex to bind, and iTreg cells generally fail to fully demethylate the TSDR, a lack of binding of this complex may account for their reduced stability. CNS2 is demethylated in GFP^+^ *Foxp3*-null T cells (T_FN_) expressing a *Foxp3* reporter “null” allele (*Foxp3^*gfpko*^*), suggesting that Foxp3 binding is not required for demethylation of the TSDR (Zheng et al., 2010). Rather, it appears that TCR stimulation is essential to establish the Treg cell-specific CpG hypomethylation patterns (Ohkura et al., 2012). The last CNS element observed, CNS3, is important for Foxp3 induction in the thymus and periphery. Formation of a c-Rel containing enhanceosome, in cooperation with NFAT, CREB, p65, and Smad3, may potentiate Foxp3 induction (Rudensky, 2011). In addition to the demethylation pattern observed in CNS2 of *Foxp3*, three other “Treg cell-representative regions” were identified and included regions of *Tnfrsf18*, *Ctla4*, and *Ikzf4* that display distinct demethylation patterns in nTreg, Tconv, and iTreg cells and are essential to establish lineage stability (Ohkura et al., 2012). In addition to its roles in iTreg cell generation and proliferation, IL-2 signaling is important for iTreg cell stability *in vivo* (Chen et al., 2011). Also, expression of the suppressor of cytokine signaling-2 (SOCS2) protein plays a role in preventing IL-4-dependent iTreg instability (Knosp et al., 2013). In summary, a complex, regulated series of interactions with Foxp3 are required for the establishment of Treg cell stability.

**Table 2:** **Summary of CNS2 methylation status in CD4^+^ T cell populations**.

Reference	Cell type	Method	% methylation
Floess et al. (2007)	nTreg	CD4^+^CD8^−^CD25^+^ Treg cells isolated from the thymus	+++
	nTreg	CD25^+^ Treg cells isolated from secondary lymphoid organs of male mice	+
	iTreg	Mouse TGF-β1 induced iTreg cells after 6 days in culture	+++
	Tconv	CD25^−^CD4^+^ Tconv cells isolated from secondary lymphoid organs of male mice	++++
Baron et al. (2007)	nTreg	Human FOXP3^+^CD25^*high*^CD4^+^ Treg cells isolated from the peripheral blood of male donors	+
	Tconv	Human naive CD45RA^+^CD25^−^CD4^+^ T cells isolated from the peripheral blood of male donors	++++
Polansky et al. (2008)	iTreg	iTreg cells generated *in vivo* by anti-DEC-205-mediated targeting of an agonist to dendritic cells, isolated 3 weeks later and expanded *in vitro* for 5 days	++
Lal et al. (2009)	iTreg	iTreg cells generated in the presence of TGF-β1 and the DNA methyltransferase inhibitor 5-aza-2′-deoxycytidine	+
Zheng et al. (2010)	nTreg	GFP^+^ Foxp3-null T cells (T_FN_) expressing a *Foxp3* reporter “null” allele (*Foxp3^*gfpko*^*)	+
	iTreg	TGF-β1 induced iTreg cells after 3 days in culture	++++
Haribhai et al. (2011)	nTreg	nTreg cells transferred into a Foxp3-deficient host at birth and maintained *in vivo* 50 days	+
	iTreg	Generated *in vivo* from Tconv cells that were transferred into Foxp3-deficient mice at birth and maintained *in vivo* 50 days	++++
Chen et al. (2011)	iTreg	Transfer of OT-II iTreg cells followed by immunization with OVA/IFA and treatment with IL-2/anti-IL-2 complexes, isolated after 5 days	+
Sela et al. (2011)	iTreg	Generated by a MLR in the presence of TGF-β1 and RA, cultured 5 days	+++
	iTreg	Generated by a MLR in the presence of TGF-β1 and RA, cultured 5 days and restimulated with allogeneic dendritic cells for 3 days	++
	iTreg	Generated *in vitro* by a 5-day MLR in the presence of TGF-β1 and RA, cotransferred with GVHD-inducing cells, and isolated 1.5 months post transfer	+
Ohkura et al. (2012)	nTreg	Isolated from the thymus	+++
	nTreg	Isolated from the spleen	+
	iTreg	Generated *in vitro* by TCR stimulation with TGF-β1 ± RA, 5 days culture	++++
	iTreg	Transfer of Tconv into RagKO recipients, analysis of *in vivo*-derived iTreg cells 7 weeks post transfer	+
Miyao et al. (2012)	Tconv	*In vitro* TCR stimulation of naïve T cells in the presence of IL-2, leading to transient activation induced Foxp3 expression	++++
Schmitt et al. (2012)	nTreg	nTreg cells used to treat lymphopenia induced colitis, maintained *in vivo ∼*100 days	+
	iTreg	Generated *in vitro* with TGF-β1, maintained *in vivo* (as above) for ∼100 days	+++
Toker et al. (2013)	nTreg	Thymic CD4^+^CD8^−^Foxp3^+^CD24^*hi*^	++++
	nTreg	Thymic CD4^+^CD8^−^Foxp3^+^CD24^*int*^	+++
	nTreg	Thymic CD4^+^CD8^−^Foxp3^+^CD24^*lo*^	++

*This table documents the percent methylation of CNS2, also known as the Treg cell-specific demethylated region (TSDR), of nTreg, iTreg, and Tconv cells in several different model systems and organs. Percent methylation is depicted as follows: +, 0–25% methylated; ++, 25–50% methylated; +++, 50–75% methylated; ++++, 75–100% methylated. GVHD, graft versus host disease; IFA, incomplete Freud’s adjuvant; MLR, mixed leukocyte reaction; OVA, ovalbumin; RA, retinoic acid*.

## Conclusion

In conclusion, recent work has established the importance of iTreg cells to the maintenance of immunological tolerance. As a population, iTreg cells share many characteristics with nTreg cells, but the observed differences in their respective TCR repertoires may lead to differential function and location, creating a need for both subsets. Future studies will look to establish additional surface markers to distinguish the subsets so that conclusive studies regarding the function and stability of the iTreg cell population can be conducted. An enhanced understanding of the origin and function of iTreg cells will promote future studies examining the translational potential of these cells.

## Conflict of Interest Statement

The authors declare that the research was conducted in the absence of any commercial or financial relationships that could be construed as a potential conflict of interest.
